# The *adc1* knockout with *proC* overexpression in *Synechocystis* sp. PCC 6803 induces a diversion of acetyl-CoA to produce more polyhydroxybutyrate

**DOI:** 10.1186/s13068-024-02458-9

**Published:** 2024-01-13

**Authors:** Suthira Utharn, Saowarath Jantaro

**Affiliations:** 1 Laboratory of Cyanobacterial Biotechnology, Department of Biochemistry, Faculty of Science, Chulalongkorn University, Bangkok, 10330 Thailand; 2 Program of Biotechnology, Faculty of Science, Chulalongkorn University, Bangkok, 10330 Thailand

**Keywords:** PHB, *Synechocystis* sp. PCC 6803, Proline, Glycogen, Glutamate

## Abstract

**Background:**

Lack of nutrients, in particular nitrogen and phosphorus, has been known in the field to sense glutamate production via 2-oxoglutarate and subsequently accelerate carbon storage, including glycogen and polyhydroxybutyrate (PHB), in cyanobacteria, but a few studies have focused on arginine catabolism. In this study, we first time demonstrated that gene manipulation on *proC* and *adc1*, related to proline and polyamine syntheses in arginine catabolism, had a significant impact on enhanced PHB production during late growth phase and nutrient-modified conditions. We constructed *Synechocystis* sp. PCC 6803 with an overexpressing *proC* gene, encoding Δ^1^pyrroline-5-carboxylate reductase in proline production, and *adc1* disruption resulted in lower polyamine synthesis.

**Results:**

Three engineered *Synechocystis* sp. PCC 6803 strains, including a *ProC*-overexpressing strain (OXP), *adc1* mutant, and an OXP strain lacking the *adc1* gene (OXP/Δ*adc1*), certainly increased the PHB accumulation under nitrogen and phosphorus deficiency. The possible advantages of single *proC* overexpression include improved PHB and glycogen storage in late phase of growth and long-term stress situations. However, on day 7 of treatment, the synergistic impact created by OXP/Δ*adc1* increased PHB synthesis by approximately 48.9% of dry cell weight, resulting in a shorter response to nutrient stress than the OXP strain. Notably, changes in proline and glutamate contents in engineered strains, in particular OXP and OXP/Δ*adc1*, not only partially balanced the intracellular C/N metabolism but also helped cells acclimate under nitrogen (N) and phosphorus (P) stress with higher chlorophyll *a* content in comparison with wild-type control.

**Conclusions:**

In *Synechocystis* sp. PCC 6803, overexpression of *proC* resulted in a striking signal to PHB and glycogen accumulation after prolonged nutrient deprivation. When combined with the *adc*1 disruption, there was a notable increase in PHB production, particularly in situations where there was a strong C supply and a lack of N and P.

**Supplementary Information:**

The online version contains supplementary material available at 10.1186/s13068-024-02458-9.

## Introduction

**Fig. 1:**
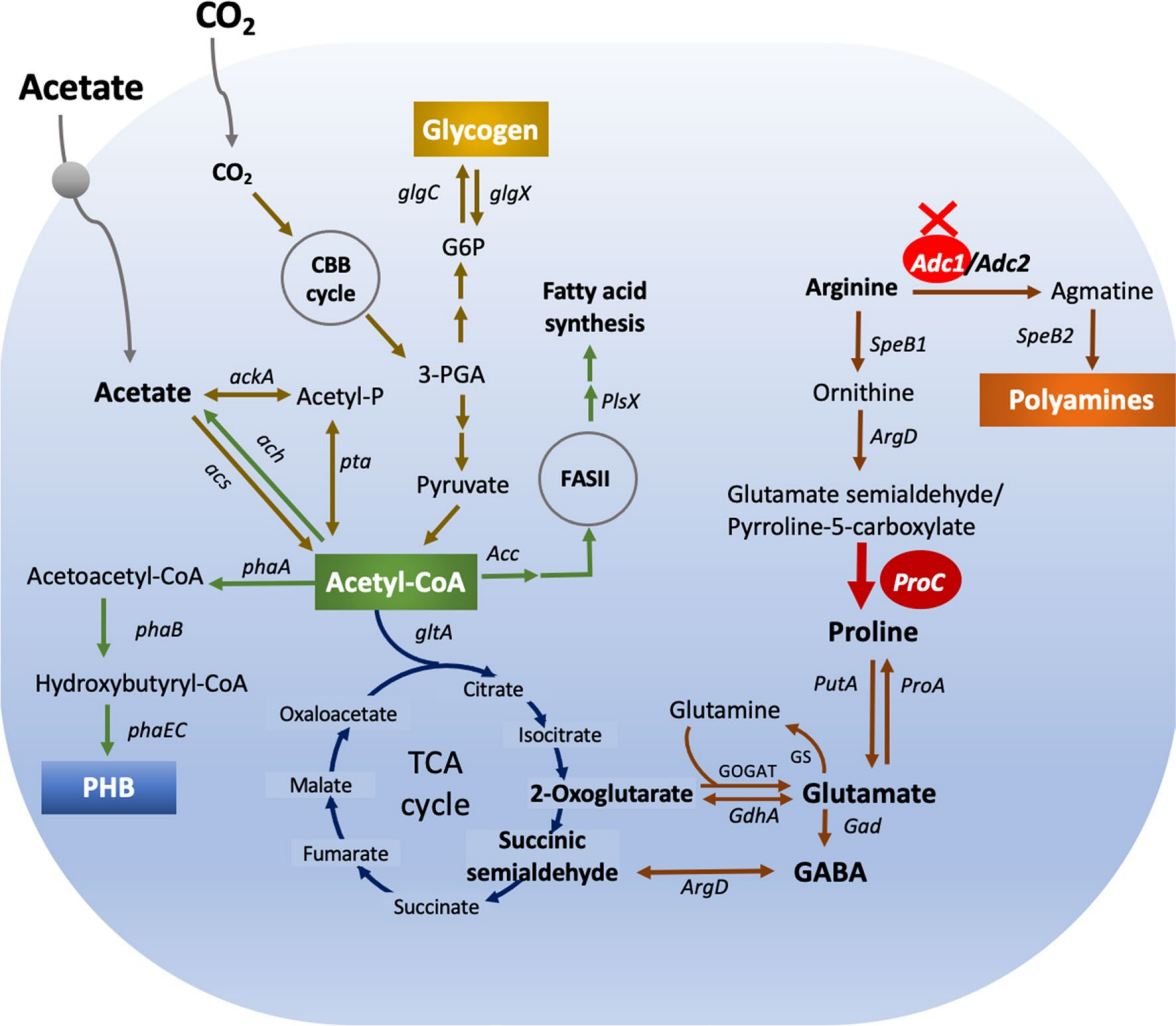
Overview of the polyamine-proline-glutamate pathways connected to the tricarboxylic acid (TCA) cycle and related biosynthetic pathways of polyhydroxybutyrate (PHB), and glycogen in cyanobacterium *Synechocystis* sp. PCC 6803. Abbreviations of genes are: *acc*, a multisubunit acetyl-CoA carboxylase; *ach*, acetyl-CoA hydrolase; *ackA*, acetate kinase; *acs*, acetyl-CoA synthase; *adc*, arginine decarboxylase; *argD*, N-acetylornithine aminotransferase; *gad*, glutamate decarboxylase; *gdhA*, glutamate dehydrogenase; *glgC*, ADP-glucose pyrophosphorylase; *glgX*, glycogen debranching enzyme; *gltA*, citrate synthase; *plsX*, fatty acid/phospholipid synthesis protein; *phaA*, β-ketothiolase; *phaB*, acetoacetyl-CoA reductase; *phaC* and *phaE*, the heterodimeric PHB synthase; *proA*, gamma-glutamyl phosphate reductase; *proC*, Δ^1^pyrroline-5-carboxylate reductase; *pta*, phosphotransacetylase; *putA*, proline oxidase; *speB1*, arginase; *speB2*, agmatinase. Abbreviations of intermediates are: FASII, fatty acid synthesis type II; GABA, gamma-aminobutyric acid; GOGAT, glutamate synthase; G6P, glucose-6-phosphate; GS, glutamine synthetase; 3-PGA, 3-phosphoglycerate; PHB, polyhydroxybutyrate

The cumulative harm caused by pollution and inadequate resource and land management of our remaining natural resources is a major connected element of many global concerns. Microalgae, including cyanobacteria, have the ability to address some of these issues by decreasing aquatic pollutants and offering a sustainable supply of biomass for product development, as evidenced by the emerging uses of microalgal biotechnology assisting the United Nations’ Sustainable Development Goals (SDGs) [1, 2]. The microalgal biomass possesses a wide variety of primary and secondary metabolites that are increasingly being acknowledged for their importance in the production of novel products and biotechnological applications. These valuable products, which can be produced directly from CO_2_ via photosynthesis, include pigments such as carotenoids, and chlorophylls, carbon storages such as glycogen and polyhydroxybutyrate (PHB), as well as macromolecular compounds such as proteins, carbohydrates, and lipids [3–9]. There are several strategies for boosting algal biomass in order to achieve the lower carbon restriction that limits the yield of biofuel and bioproducts, including nutritional adjustment, and gene manipulation by genetic and metabolic engineering. The increased CO_2_ fixation capacity, such as *RuBisCO* gene overexpression, and glucose utilization, has driven cyanobacteria to have better growth and photosynthesis [6, 10–12], and be able to produce more PHB and lipids [4]. Under nitrogen or phosphorus deficiency, most cyanobacteria preferentially store carbon sources in the forms of glycogen and PHB, which are a consequence of the 2-oxoglutarate balance for carbon/nitrogen control [13–15]. In Fig. 1, the arginine catabolism flux associated with the polyamine synthesis, proline-glutamate reaction, and GS/GOGAT pathway [16, 17]. Previous studies found that a transposon-mutated *Synechocystis* sp. PCC 6803 significantly enhanced PHB synthesis while lacking the *proA* gene encoding gamma-glutamyl phosphate reductase. This mutant also had decreased proline reduction and increased glutamate accumulation [18]. Furthermore, the arginine–ornithine to proline–glutamate reaction was primarily driven by the deletion of the *adc1* gene, which encodes arginine decarboxylase involved in polyamine biosynthesis, in *Synechocystis* sp. PCC 6803. This undoubtedly boosted PHB production, although the precise mechanism is still unknown [5]. Notably, in *Synechocystis* sp. PCC 6803, during nitrogen shortage, the activation of an OmpR-type response regulator (Rre37) may stimulate the metabolic flux from glycogen to PHB as well as the hybrid TCA cycle and arginine–ornithine cycle [9]. In cyanobacteria, glutamate can be synthesized through two alternative systems including GS/GOGAT pathway, and another catalyzed by glutamate dehydrogenase (GDH) [19, 20]. The GS/GOGAT pathway is the main ammonium assimilation system in *Synechocystis* 6803, while GDH encoded by the *gdhA* gene is regulated by the late stage of growth [21, 22] and when energy supply is limited in *Escherichia coli* [23]. On the other hand, when acetyl-CoA flow is driven to PHB accumulation, it is mostly directed to the TCA cycle and fatty acid synthesis from pyruvate and acetate, except for nutritional constraints (Fig. 1). Enzymes which involved in PHB biosynthesis are β-ketothiolase (phaA), acetoacetyl-CoA reductase (phaB), and heterodimeric PHB synthase (phaE and phaC), respectively [24]. Instead of producing new CO_2_ fixation, the cyanobacteria that were starved of nutrients favored producing PHB from internally stored carbon storage, such as glycogen [8, 25]. In this study, to supply more glutamate to TCA cycle, we overexpressed the *proC* gene, encoding Δ^1^pyrroline-5-carboxylate reductase (Fig. 1), in *Synechocystis* sp. PCC6803 wild type and Δ*adc1* mutant strains. Two engineered strains included a *Synechocystis* sp. PCC6803 overexpressing *proC* gene or OXP, and *Synechocystis* sp. PCC6803 overexpressing *proC* gene with a knockout of *adc1* gene in polyamine synthesis or OXP/Δ*adc1* strains. Both engineered strains certainly accumulated higher PHB content, particularly in a nitrogen and phosphorus-deprived BG_11_ medium with acetate supplementation (BG_11_-N-P + A). It is important to highlight that, particularly in the presence of BG_11_-N-P + A, the acetyl-CoA flow was mainly diverted to the PHB biosynthetic pathway.

## Results

### Overexpression of native *proC* gene in *Synechocystis* sp. PCC 6803 wild-type and mutant strains

**Fig. 2:**
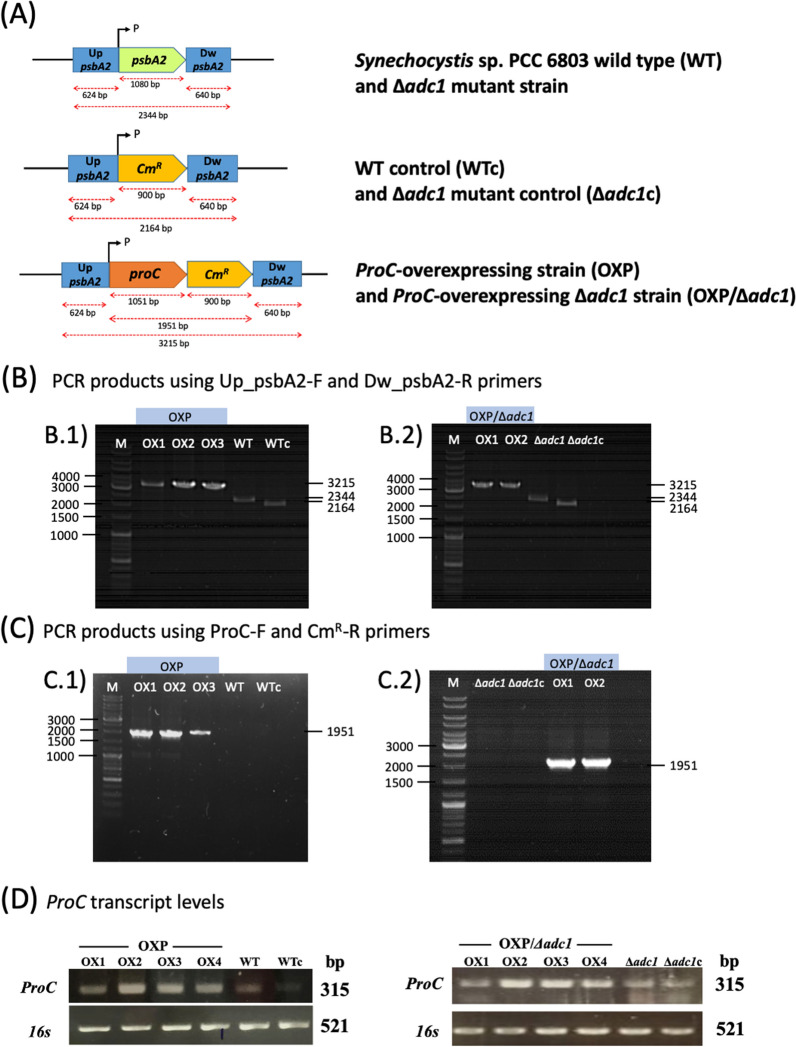
Genomic maps and transcript levels of *Synechocystis* sp. PCC 6803 strains. The four constructed strains are *Synechocystis* sp. PCC 6803 wild-type control (WTc), *Synechocystis* sp. PCC 6803 lacking *adc1* gene (Δ*adc1*c), *Synechocystis* sp. PCC 6803 overexpressing *proC* gene (OXP), and Δ*adc1* mutant overexpressing *proC* gene (OXP/Δ*adc1*). PCR analysis employing two pairs of specific primers (Supplementary information Table S1) was used to confirm the accurate integration and placement of each gene fragment into the *Synechocystis* genome. (**A**) The double homologous recombination of both *Cm*^*R*^ gene occurred between the conserved sequences of *psbA2* gene in WTc and Δ*adc1*c, and a *proC*:*Cm*^*R*^ fragment occurred between the conserved sequences of *psbA2* gene in OXP and OXP/Δ*adc1* strains when compared to WT. (**B**) For PCR products using UP_psbA2-F and Dw_psbA2-R primers, (**B.1**) For OXP strain, Lane M: GeneRuler DNA ladder, Lanes OX1, OX2, and OX3: three clones no. 1–3 containing a 3.2 kb fragment of Up_*psbA2*-*proC-Cm*^*R*^-Dw_*psbA2*, Lanes WT and WTc: negative controls of a 2.4 kb fragment in WT and a 2.2 kb fragment in WTc, respectively. (**B.2**) For OXP/Δ*adc1* strain, Lane M: GeneRuler DNA ladder, Lanes OX1 and OX2: two clones no. 1 and 2 containing a 3.2 kb fragment of Up_*psbA2*-*proC-Cm*^*R*^-Dw_*psbA2*, Lanes Δ*adc1* and Δ*adc1*c: negative controls of a 2.4 kb fragment in Δ*adc1* and a 2.2 kb fragment in Δ*adc1*c, respectively. (**C**) For PCR products using ProC-F and Cm^R^-R primers, (**C.1**) For OXP strain, Lane M: GeneRuler DNA ladder, Lanes OX1, OX2, and OX3: three clones no. 1–3 containing a 1.9 kb fragment of *proC-Cm*^*R*^, Lanes WT and WTc: negative controls (no band) using WT and WTc as template, respectively. (**C.2**) For OXP/Δ*adc1* strain, Lane M: GeneRuler DNA ladder, Lanes Δ*adc1* and Δ*adc1*c: negative controls (no band) using Δ*adc1* and Δ*adc1*c as template, respectively, Lanes OX1 and OX2: two clones no. 1 and 2 containing a 1.9 kb fragment of *proC-Cm*^*R*^. (**D**) Transcript levels of *proC* gene determined by RT-PCR using RT-ProC-F and RT-ProC-R primers (Additional file 1: Table S1) in WT, WTc, Δ*adc1*, Δ*adc1*c, and two overexpressing strains, including OXP and OXP/Δ*adc1.* The 0.8% agarose gel electrophoresis of PCR products was performed from cells grown for 6 days in normal BG_11_ medium. The *16s* rRNA was used as reference. The cropped gels (in **D**) were taken from the original images of RT-PCR products on agarose gels as shown in Supplementary information Figure S1

Initially, we constructed four engineered *Synechocystis* sp. PCC 6803 strains, including wild-type control (WTc), Δ*adc1* mutant control (Δ*adc1*c), OXP, and OXP/Δ*adc1* by double homologous recombination (Table 1, Fig. 2A). For the WTc and Δ*adc1*c strains, they were created by replacing the *psbA2* gene with a *Cm*^*R*^ cassette in the genomes of *Synechocystis* sp. PCC 6803 WT and Δ*adc1* mutant, respectively (Fig. 2A). To create a recombinant plasmid pEERM_*proC* (Table 1), a native *proC* (or *slr0661*) gene fragment with a size of 1.0 kb was ligated between flanking regions of the *psbA2* gene of the pEERM vector and the upstream region of *Cm*^*R*^ cassette (Fig. 2A). Next, all overexpressing strains were verified by PCR using specific pairs of primers (Additional file 1: Table S1) for their complete segregation and gene location. To confirm the complete segregation, PCR products with Up_psbA2-F and Dw_psbA2-R primers confirmed the correct size of 3.2 kb, in OXP and OXP/Δ*adc1* strains (Fig. 2B.1 and B.2, respectively), while there were 2.3 kb in WT and Δ*adc1* strains, and 2.2 kb in WTc and Δ*adc1*c strains. PCR products with ProC-F and Cm^R^-R primers confirmed the correct size of 1.9 kb in OXP and OXP/Δ*adc1* strains (Fig. 2C1 and C2, respectively), compared with no band in WT, WTc, Δ*adc1* and Δ*adc1*c strains. In addition, *ProC* gene overexpression was verified by RT-PCR data in all engineered strains (Fig. 2D).

**Table 1:** Strains and plasmids used in this study

Name	Relevant genotype	References
Cyanobacterial strains		
*Synechocystis* sp. PCC 6803	Wild type	Pasteur culture collection
Δ*adc1*	Δ*adc1* knockout, *Km*^*R*^ inserted between *adc1* gene in *Synechocystis* genome	[5]
WT control (WTc)	WT, *Cm*^*R*^ integrated at flanking region of *psbA2* gene in *Synechocystis* genome	This study
Δ*adc1* control (Δ*adc1*c)	Δ*adc1*, *Cm*^*R*^ integrated at flanking region of *psbA2* gene in *Synechocystis* genome	This study
OXP	*ProC*, *Cm*^*R*^ integrated at flanking region of *psbA2* gene in *Synechocystis* WT genome	This study
OXP/Δ*adc1*	*ProC*, *Cm*^*R*^ integrated at flanking region of *psbA2* gene in *Synechocystis* Δ*adc1* genome	This study
Plasmids		
pEERM	P_psbA2_- *Cm*^*R*^; plasmid containing flanking region of *psbA2* gene	[26]
pEERM_*ProC*	P_psbA2_-*ProC*- *Cm*^*R*^; integrated between *Spe*I and *Pst*I sites of pEERM	This study

P_psbA2_, *psbA2* promoter; *Cm*^*R*^, chloramphenicol resistance cassette

### Growth, intracellular pigment contents, oxygen evolution rates, and metabolite accumulation under normal growth condition

**Fig. 3:**
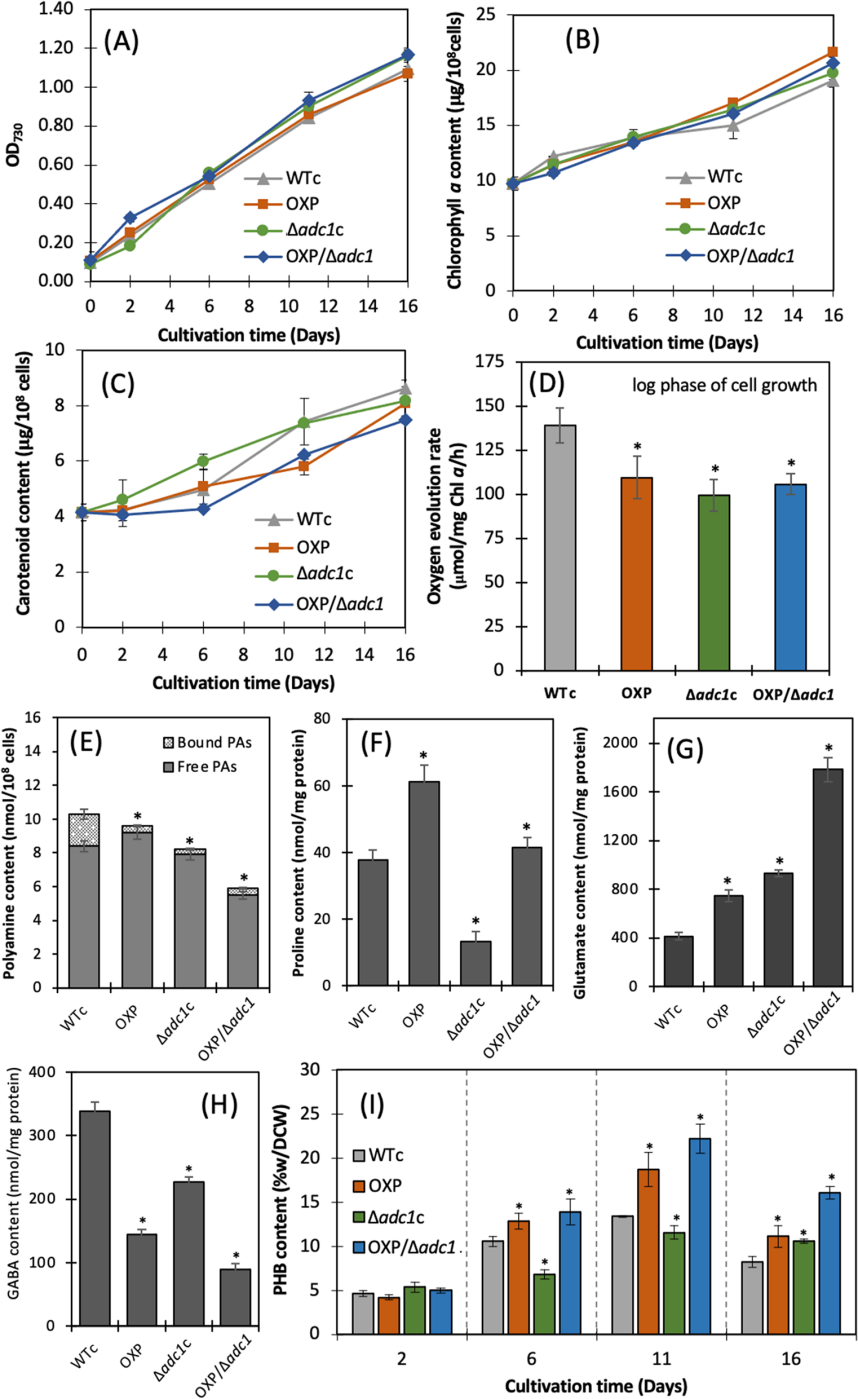
Growth curve (**A**), chlorophyll *a* content (**B**), carotenoid content (**C**), oxygen evolution rate (**D**), contents of polyamines (**E**), proline (**F**), glutamate (**G**), GABA (**H**), and PHB (**I**) of WTc, Δ*adc1*c, OXP, and OXP/Δ*adc1 Synechocystis* sp. PCC 6803 strains. In (**A**–**C**), and (**I**), cells grown in BG_11_ medium for 16 days. In (**D**–**H**), cells were grown in normal BG_11_ medium for 7 days, and harvested for metabolite contents. The error bars represent standard deviations of means (mean ± S.D., *n* = 3). In (**D**–**I**), the statistical difference of the results between those values of WTc and that engineered strain is indicated by an asterisk at *P < 0.05

We found a slight increase in cell growth of the Δ*adc1*c and OXP/Δ*adc1* strains in comparison with the WTc and OXP strains (Fig. 3A). All strains had comparable amounts of chlorophyll *a*; however, *proC* overexpression in OXP and OXP/Δ*adc1* had an impact on the decrease of carotenoids (Fig. 3B, C). In comparison to WTc, all engineered strains showed lower rates of oxygen evolution (Fig. 3D). On the other hand, as anticipated, total polyamines (PAs) in both bound and free forms declined in the Δ*adc1*c and OXP/Δ*adc1* strains, but the OXP strain showed a minor decrease in total PAs as compared to the WTc strain (Fig. 3E). It was found that bound PAs were the main decrease when the *adc1* gene was disrupted. On day 7 under normal growth condition, the proline levels of OXP and OXP/Δ*adc1* strains were found to be much higher, but the Δ*adc1*c strain has the lowest proline content (Fig. 3F). Moreover, the WTc strain exhibited significant glutamate content that was almost ten times greater than proline under normal growth conditions (Fig. 3G). All mutant strains, especially the OXP/Δ*adc1* strain, had a greater increase in glutamate content than the WTc. Similarly, on day 7 of culture, the GABA level in WTc was higher than the proline content but somewhat lower than the glutamate content under normal condition (Fig. 3H). The GABA content of all engineered strains, especially OXP/Δ*adc1*, was lower than that of the WTc strain. Glutamate appeared to be the preferred compound that *Synechocystis* cells accumulated, followed by GABA and proline. On the other hand, cells substantially produced a low level of PHB by about 4 – 23% w/DCW under normal growth condition (Fig. 3I). When compared to the WTc, the PHB quantity in the OXP and OXP/Δ*adc1* strains appeared to be larger. In order to adapt to the nutrient-modified medium, cells growing on day 11, which represents the late-log phase of cell growth with the maximum level of PHB accumulation, were subsequently selected.

### Growth, intracellular pigment contents, and metabolite accumulation under nutrient-modified conditions

**Fig. 4:**
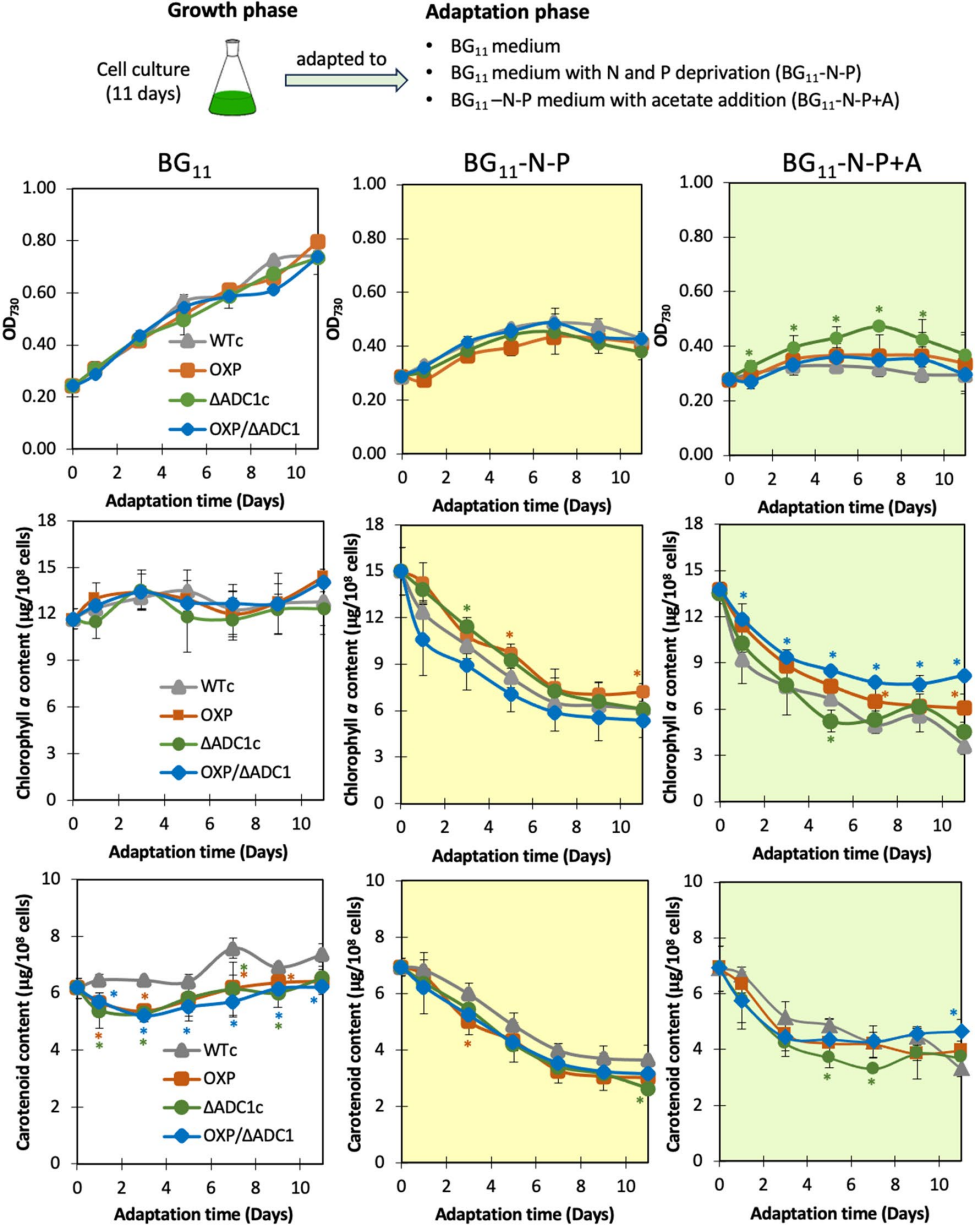
Growth curve (**A**–**C**), chlorophyll *a* content (**D**–**F**), and carotenoid content (**G**–**I**) of *Synechocystis* WTc, Δ*adc1*c, OXP, and OXP/Δ*adc1* strains adapted in normal BG_11_ medium, BG_11_ medium with N and P deprivation (BG_11_-N-P), and BG_11_-N-P supplemented with 4%(w/v) acetate (BG_11_-N-P + A) medium for 11 days. The error bars represent standard deviations of means (mean ± S.D., *n* = 3). The statistical difference of the results between those values of WTc and that engineered strain is indicated by an asterisk at *P < 0.05

All *Synechocystis* strains were grown in normal BG_11_ medium for 11 days before starting the adaptation phase (Fig. 4). Both two nutrient-modified media, including BG_11_ lacking nitrogen and phosphorus (BG_11_-N-P) and BG_11_-N-P medium with acetate addition (BG_11_-N-P + A), caused a certain reduction in growth (Fig. 4A–C) and intracellular contents of chlorophyll *a* and carotenoids (Fig. 4D–I). It is worth noting that all engineered strains had a slightly higher level of cell growth under the BG_11_-N-P + A condition, in particular Δ*adc1*c (Fig. 4C). In addition, the *proC*-overexpressing strains, including OXP and OXP/Δ*adc1*, contained a higher accumulation of chlorophyll a than WTc under the BG_11_-N-P + A condition (Fig. 4F).

**Fig. 5:**
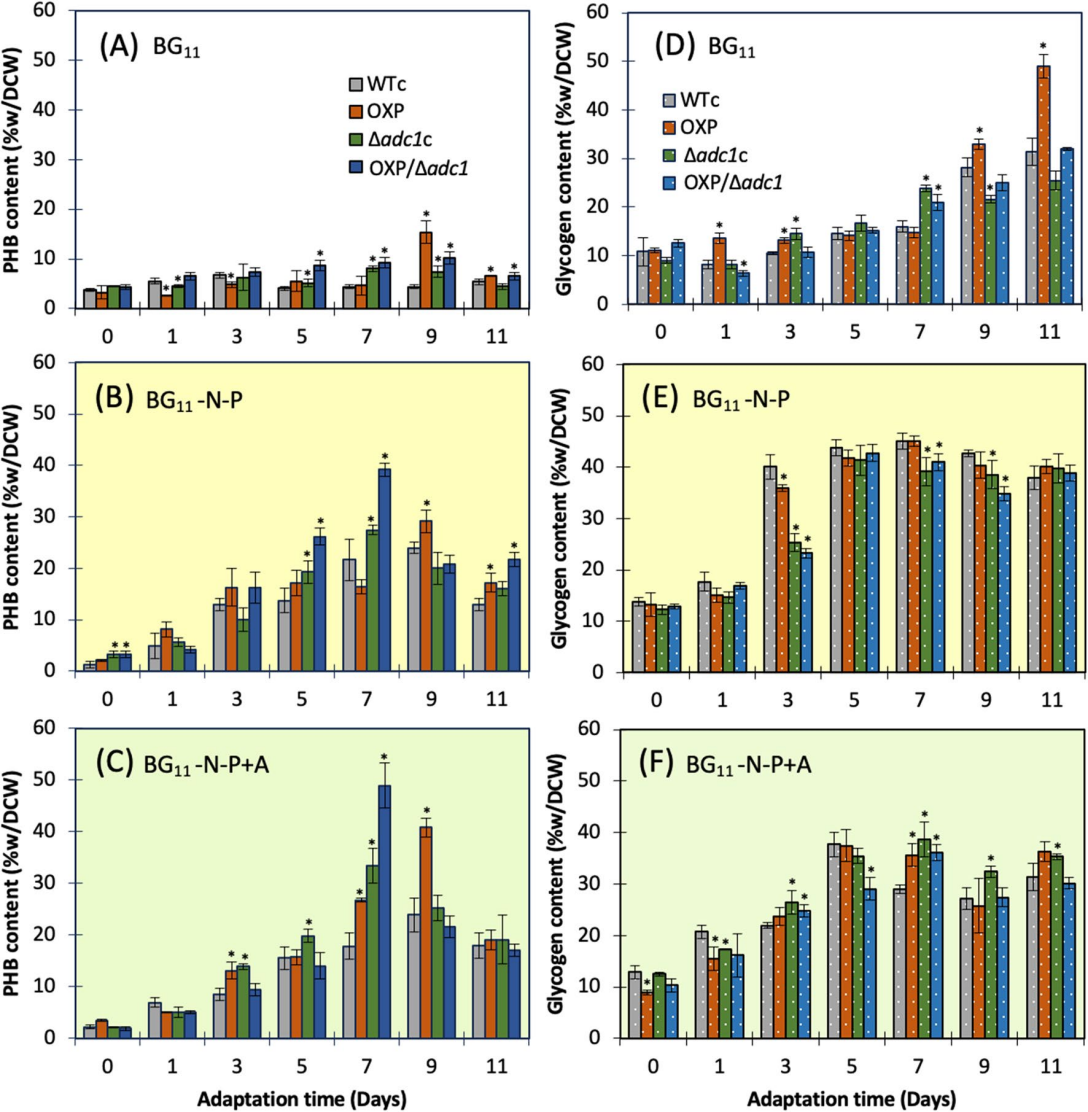
Contents of PHB (**A**–**C**) and glycogen (**D**–**F**) of *Synechocystis* WTc, Δ*adc1*c, OXP, and OXP/Δ*adc1* strains adapted in normal BG_11_ medium, BG_11_ medium with N and P deprivation (BG_11_-N-P), and BG_11_-N-P supplemented with 4%(w/v) acetate (BG_11_-N-P + A) medium for 11 days. The error bars represent standard deviations of means (mean ± S.D., *n* = 3). An asterisk (*P < 0.05) denotes the statistical difference in results between those WTc values and that engineered strain at each day

**Fig. 6:**
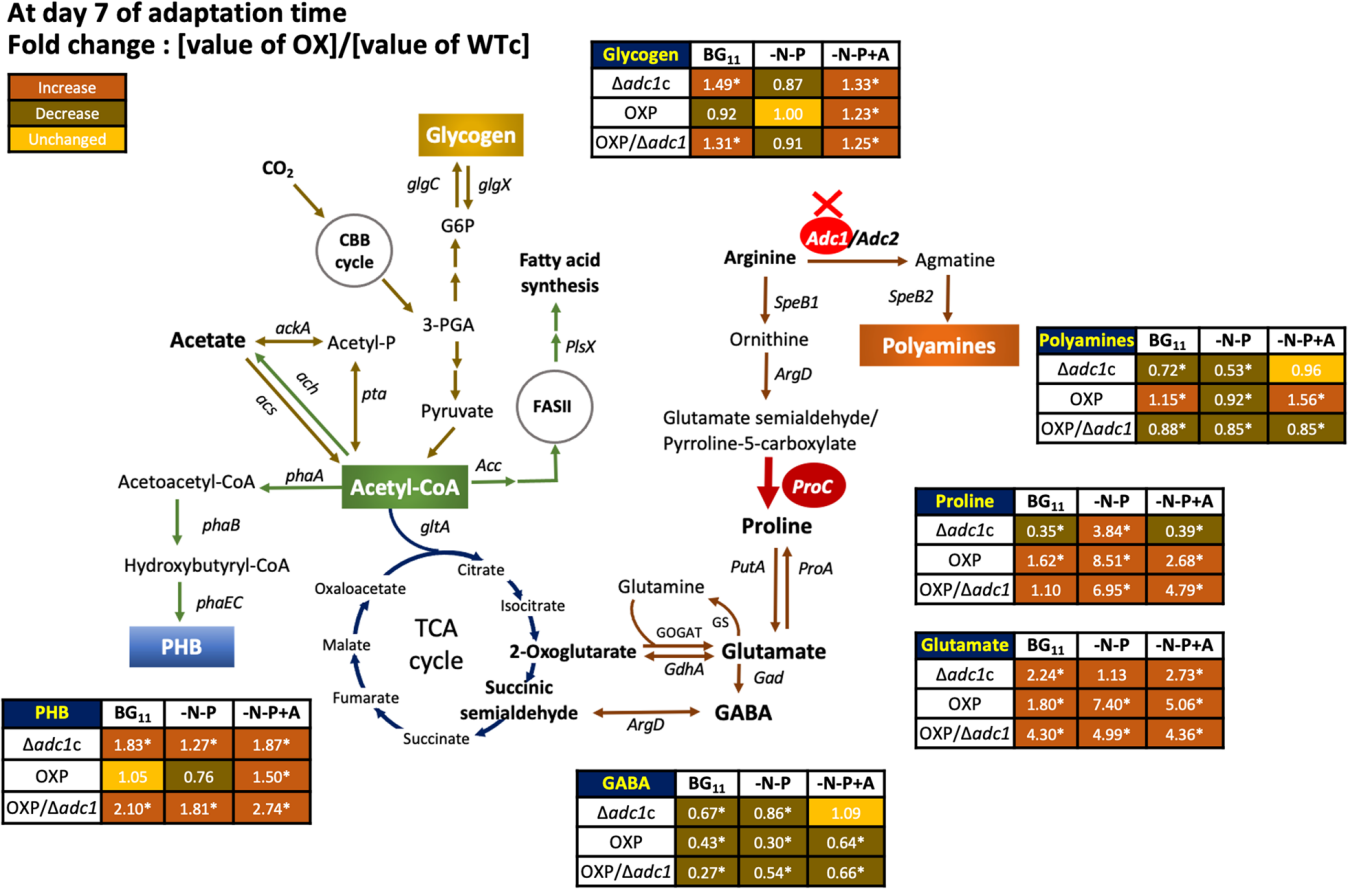
Fold changes of obtained results of metabolite products in three engineered strains compared with those in *Synechocystis* WTc after adapting cells in BG_11_, BG_11_-N-P, and BG_11_-N-P + A for 7 days. In each box, the number represents the fold change of that value of each engineered strain under each stress condition divided by that value of WTc. The statistical difference in the data between those values of WT and the engineered strain is represented by an asterisk at *P < 0.05

For the main carbon storages of glycogen and polyhydroxybutyrate (PHB) (Fig. 5), glycogen was markedly accumulated rather than PHB under normal growth condition, in particular for a longer period at days 9–11 of cultivation (Fig. 5A and D). The OXP strain contained the highest level of glycogen, up to 30–49% of dry cell weight, among other strains, during 9–11 days under normal BG_11_ condition (Fig. 5D). Both BG_11_-N-P and BG_11_-N-P + A conditions promoted the specific induction of PHB synthesis in all strains, whereas cells comparatively reduced the quantity of glycogen compared to those under normal condition (Fig. 5B–F). Remarkably, on day 7 of the adaptation phase of both BG_11_-N-P and BG_11_-N-P + A media, the OXP/Δ*adc1* strain accumulated the greatest amount of PHB, with around 39.2 and 48.9%w/DCW, respectively (Fig. 5B, C). Moreover, in Fig. 6, there was a 2.7-fold increase in PHB in the OXP/Δ*adc1* strain compared to WTc at day 7 under a BG_11_-N-P + A condition. Interestingly, after adapting to both BG_11_-N-P and BG_11_-N-P + A media, the PHB accumulation of the OXP strain was later driven to reach its maximum level on day 9. On the other hand, it was anticipated that these BG_11_-N-P and BG_11_-N-P + A conditions would result in a reduction in polyamines in all strains, particularly OXP/Δ*adc1* strain (Table 2). It was evident from Fig. 6 that the OXP/Δ*adc1* strain has decreased by 0.8 fold in comparison to WTc (Fig. 6). Regarding the proline-glutamate-GABA pathway, glutamate production predominated under typical BG_11_ condition, particularly in OXP and OXP/Δ*adc1* strains, followed by GABA and proline (Table 2). The proline content was presumably increased by BG_11_-N-P and BG_11_-N-P + A conditions, based on the greater fold change compared to WTc in OXP and OXP/Δ*adc1* strains (Fig. 6). Glutamate accumulation was reduced (Table 2), but in the modified strains, specifically the OXP strain, it increased by more than 5—7 times in both BG_11_-N-P and BG_11_-N-P + A conditions, compared to the WTc (Fig. 6). Moreover, GABA accumulation was mostly decreased under nutrient-modified conditions.

**Table 2:** Contents of some metabolites related to polyamine-proline-glutamate pathway. Cells were grown in various media for 7 days (means ± S.D., n = 3). The statistical difference in the data between the values of WT and the engineered strain is represented by an asterisk at * *p* < 0.05

Metabolites/Strains	Contents		
	BG_11_ control	BG_11_-N-P	BG_11_-N-P + A
Total polyamine contents (nmol/10^8^ cells)			
WTc	12.4 ± 0.8	7.2 ± 0.2	5.4 ± 0.3
Δ*adc1*c	8.9 ± 0.6*	3.8 ± 0.4*	5.2 ± 0.4
OXP	14.3 ± 0.5*	6.6 ± 0.1*	8.4 ± 0.5*
OXP/Δ*adc1*	10.9 ± 0.2*	6.1 ± 0.2*	4.6 ± 0.1*
Proline contents (nmol/mg protein)			
WTc	37.8 ± 3.0	21.2 ± 2.0	48.5 ± 4.0
Δ*adc1*c	13.2 ± 0.9*	81.5 ± 5.0*	19.0 ± 0.6*
OXP	61.1 ± 5.4*	180.4 ± 11.0*	130.0 ± 10.0*
OXP/Δ*adc1*	41.5 ± 3.0	147.3 ± 12.0*	232.5 ± 15.0*
Glutamate contents (nmol/mg protein)			
WTc	415.2 ± 30.0	38.8 ± 3.0	58.3 ± 3.0
Δ*adc1*c	931.0 ± 20.0*	43.8 ± 3.0	159.0 ± 8.0*
OXP	747.0 ± 40.0*	287.0 ± 20.0*	294.8 ± 15.0*
OXP/Δ*adc1*	1784 ± 70.0*	193.5 ± 10.0*	253.9 ± 10.0*
GABA contents (nmol/mg protein)			
WTc	338.0 ± 10.0	294.8 ± 9.0	174.6 ± 5.0
Δ*adc1*c	226.7 ± 8.0*	253.0 ± 8.0*	190.4 ± 10.0
OXP	144.4 ± 8.0*	88.5 ± 6.0*	112.1 ± 6.0*
OXP/Δ*adc1*	89.8 ± 5.0*	159.5 ± 10.0*	115.0 ± 5.0*

**Fig. 7:**
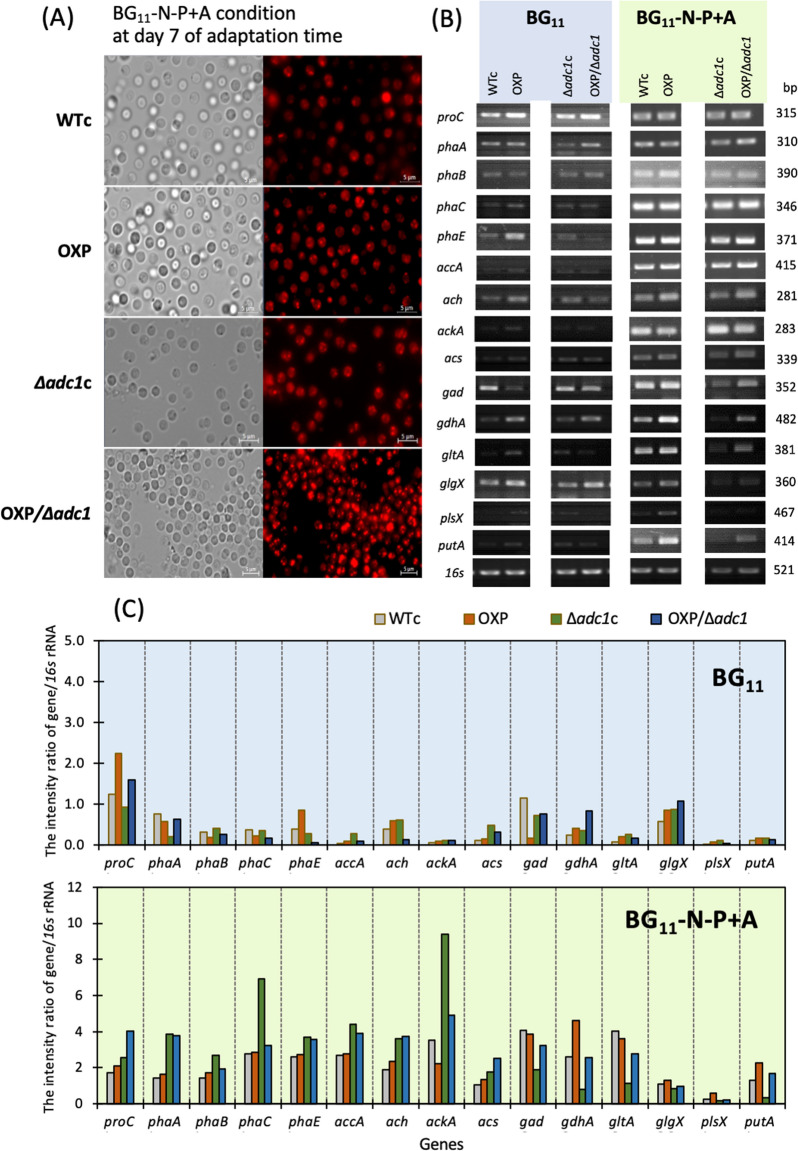
The Nile red stained PHB granules (**A**), relative transcript levels (**B**), and their band intensity ratios of gene/*16s* (**C**) of genes involved in PHB synthesis, glycogen degradation, proline-glutamate conversion, and neighboring pathways in *Synechocystis* WTc, Δ*adc1*c, OXP, and OXP/Δ*adc1* strains under BG_11_-N-P + A condition at day 7 of treatment. The *16s* rRNA was used as reference control

We also stained cells adapted under the BG_11_-N-P + A condition for 7 days with Nile red dye and visualized them under fluorescent microscopy (Fig. 7A). When compared to other strains, the OXP/Δ*adc1* strain manifestly exhibited a high abundance of PHB granules in entire cells. Furthermore, RT-PCR was conducted to measure the transcript levels of 15 different genes (Fig. 7B, C). Both in OXP and OXP/Δ*adc1* under normal and BG_11_-N-P + A conditions, the *proC* transcript level increased. It is noteworthy that OXP and OXP/Δ*adc1* strains likewise exhibited elevated *putA* transcript levels, encoding proline oxidase. Furthermore, BG_11_-N-P + A condition increased the transcript levels of the *gdhA* and *gad* genes, encoding glutamate dehydrogenase and glutamate decarboxylase, respectively, with the exception of the Δ*adc1*c strain. The transcript levels of the *acs*, *ach*, and *ackA* genes, encoding acetyl-CoA synthase, acetyl-CoA hydrolase, and acetate kinase, respectively, in acetate metabolism, were increased by the acetate supplementation in BG_11_-N-P medium. Remarkably, all strains under the BG_11_-N-P + A condition showed an increase in the transcript level of the *gltA* gene, encoding citrate synthase in a first step of the TCA cycle, when compared to the normal BG_11_ condition. On the other hand, although the BG_11_-N-P + A condition raised the quantity of the *accA* transcript, encoding acetyl-CoA carboxylase subunit A in fatty acid synthesis, relative to the normal condition, there was a low alteration in the *plsX* transcript level, encoding fatty acid/phospholipid synthesis protein. Strikingly, transcript levels of all *pha* genes, including *phaA*, *phaB*, p*haC*, and *phaE*, were upregulated by BG_11_-N-P + A condition. The reduction of *glgX* transcript amount, encoding glycogen debranching enzyme in glycogen degradation, was induced by the BG_11_-N-P + A condition rather than the normal BG_11_ control.

## Discussion

**Fig. 8:**
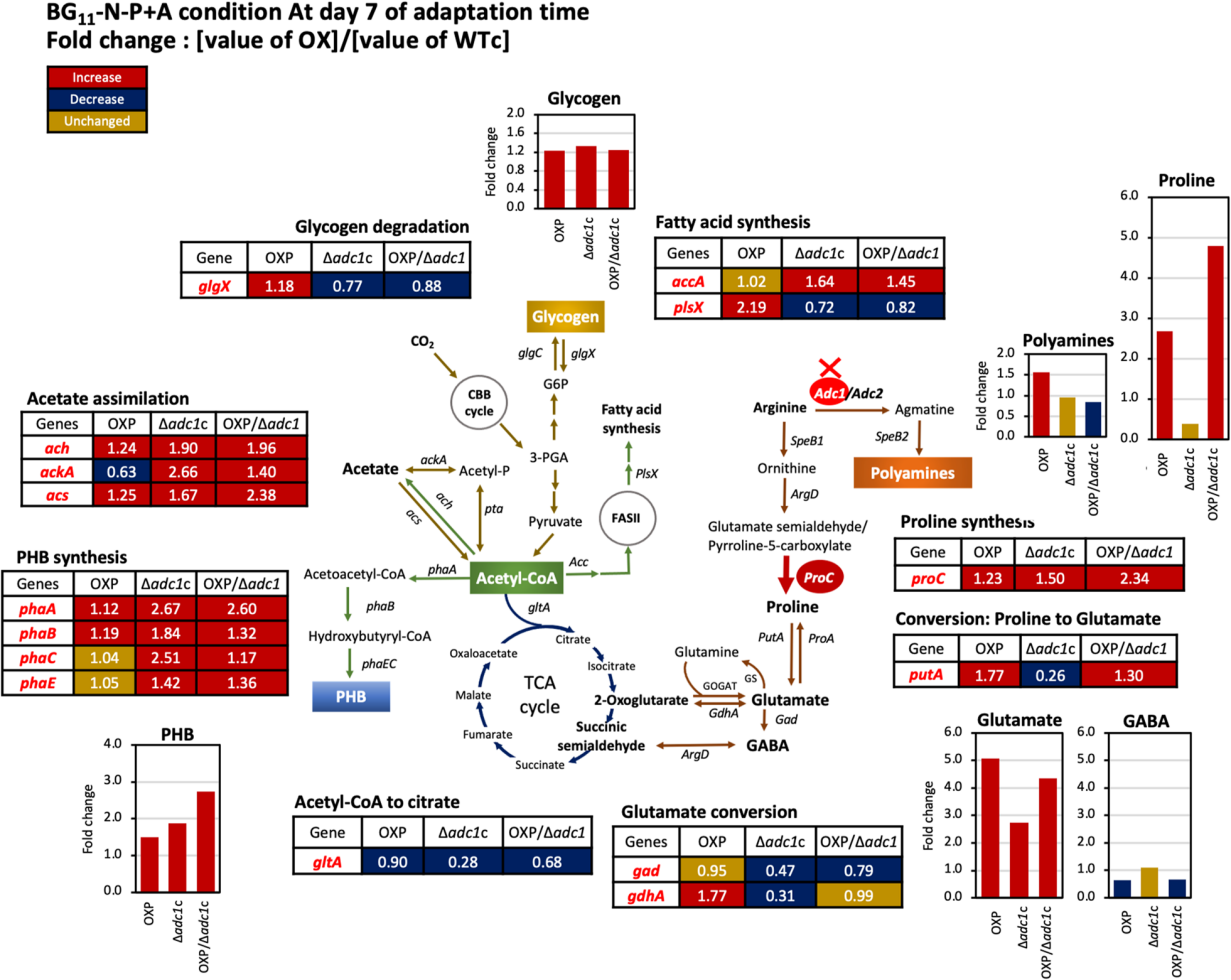
Fold changes of obtained results of gene transcript levels and metabolite contents in three engineered strains compared with those in *Synechocystis* WTc after adapting cells in BG_11_-N-P + A for 7 days. In each box and graph, the number and bar graph represents the fold change of that value of each engineered strain divided by that value of WTc, respectively

The disruption of the polyamine synthetic *adc1* gene in *Synechocystis* sp. PCC 6803 was initially discovered in a previous study [5], but the metabolic regulation remained unclear. In this work, we highlight the remarkable finding that higher PHB synthesis (up to 48.9% of dry cell weight) was caused by enhanced metabolic flux from arginine to proline and glutamate, which is closely related to nutrient stress. The introduction of the native *proC* gene, encoding pyrroline-5-carboxylate reductase of proline synthesis, in *Synechocystis* sp. PCC 6803 wild type (WT) and *adc1* mutant (Δ*adc1*) was constructed, thereby creating OXP and OXP/Δ*adc1* strains, respectively. The *proC* mutant of *Synechocystis* sp. PCC 6803 was previously shown to produce less proline, but a *putA* mutant that lacks the enzyme proline oxidase, which breaks down proline to glutamate, nonetheless accumulated a high amount of proline metabolites without producing any glutamate [16]. Enhanced proline accumulation is indicated in response to environmental stress [27–29]. In plants, the stress response of proline accumulation was controversial depending on different species and organisms; maize seedlings had an increased proline production in response to nitrogen and phosphorus deficiency [30], while French bean (*Phaseolus vulgaris* L cv Strike) plants showed a decline in proline accumulation under nitrogen-deprived condition [31], as well as a low proline level in *Arabidopsis thaliana* growing under nitrogen-limiting condition [32]. In our study, regarding BG_11_-N-P and BG_11_-N-P + A conditions, we found a minor alteration in proline accumulation in WTc as compared to BG_11_ control (Table 2). It is worthy to note that the *adc1* knockout with a decreased polyamine also contained lower proline content than the WTc, except for the BG_11_-N-P condition. Our results also indicated that glutamate accumulation in all strains was dramatically decreased in response to nitrogen and phosphorus deprivation in comparison to the WTc. Nevertheless, glutamate content increased more than twofold in all engineered strains as compared to WTc results (Fig. 8), particularly in OXP and OXP/Δ*adc1* strains. Amidst the deficiency of nitrogen and phosphorus, glutamate might have taken up a rapid key role in the metabolism of amino acids through pathways including the GS/GOGAT pathway, multispecific aminotransferases, GABA synthesis, and reversible conversions to proline and arginine [33–37]. Interestingly, the transcript level of the *gdhA* gene, encoding glutamate dehydrogenase (GDH), was upregulated only in the OXP strain which contained the highest level of glutamate under the BG_11_-N-P + A condition (Fig. 7B, C), with 1.77-fold increase compared to WTc (Fig. 8). Our finding demonstrated that the *proC* overexpression and *adc1* disruption in *Synechocystis* (OXP/Δ*adc1*) had noted results in higher proline and/or glutamate contents in comparison with WTc, which partially alleviated cells under nitrogen and phosphorus deficiency, as evidenced by the increased accumulation of chlorophyll *a*, although the stress effect of nutrient deprivation still existed.

In addition, we demonstrated that, under typical BG_11_ condition, when cells reached the late phase of growth on day 11, they accumulated more glycogen (Fig. 5D), in particular the OXP strain with 49%w/DCW, and had less PHB (Fig. 5A). The growth phase of cyanobacterial cells, in which the levels of ATP and ADP are elevated during the lag phase and then dropped during the log phase, is directly attributed to the energy charge. The improved metabolism and storage of glycogen significantly contribute to maintaining energy homeostasis [38]. Moreover, the nitrogen and phosphorus deficiency certainly accelerated the glycogen accumulation within 3 days of the adaptation phase (Fig. 5E), as well as PHB production (Fig. 5B). The impact of glycogen breakdown on PHB synthesis during nitrogen deprivation has been previously reported [8]. Subsequently, in order to improve the acetyl-CoA substrate for PHB synthesis, we added carbon source, herein acetate, to the BG_11_-N-P medium. On day 7 of the adaptation phase, there was a noticeable increase in PHB accumulation in OXP/Δ*adc1* of around 48.9% w/DCW (Fig. 5C). The increased acetate utilization in the OXP/Δ*adc1* strain in comparison to other strains confirmed this finding (Additional file 1: Fig. S2). Our results indicated that, with the exception of the OXP strain, *Synechocystis* cells favored using exogenous acetate to acetyl-CoA over glycogen breakdown, as demonstrated by a reduced amount of *glgX* transcript under BG_11_-N-P + A condition (Fig. 7B, C). Regarding acetate metabolism, cells acclimated to a BG_11_-N-P medium containing acetate exhibited significantly higher levels of the transcripts *ackA* and *acs*, encoding acetyl-CoA synthase and acetate kinase, respectively. This is consistent with their enhanced fold change, as noted in Fig. 8. It is worth noting that the OXP strain also had a higher *ackA* transcript level than that under normal BG_11_ medium (Fig. 7B), but it showed a decreased fold change when compared to WTc (Figs. 7C and 8). According to these data, the OXP strain may have utilized acetate less than other strains, which may have contributed to its reduced PHB contents on day 7 under BG_11_-N-P + A treatment compared to the Δ*adc1*c and OXP/Δ*adc1* strains. This finding was confirmed by higher amount of acetate remaining in the medium during treatment with the OXP strain than other strains (Additional file 1: Fig. S2). As demonstrated earlier by the *acs* mutant, which did not use the external acetate in medium, it is crucial to stress that the Acs enzyme functions as the primary metabolic route for acetate absorption in *Synechocystis* sp. PCC 6803 [39]. Remarkably, we suggested that the flow of acetyl-CoA metabolite to citrate in the TCA cycle in all strains was induced by the BG_11_-N-P + A condition, as supported by the upregulated transcript level of *gltA* gene, encoding citrate synthase (Fig. 7B, C). Nonetheless, it is crucial to note that the *gltA* transcript levels in the engineered strains, including OXP, *adc1*c, and OXP/Δ*adc1*, were lower than the WTc (Fig. 8). This result indicated that the lowered flow of acetyl-CoA to the TCA cycle in engineered strains in comparison to WTc substantially contributed to driving acetyl-CoA to other flux directions, such as PHB biosynthetic pathway and fatty acid synthesis. Then, we postulated that the increased levels of proline and glutamate in the engineered strains OXP, Δ*adc1*c, and OXP/Δ*adc1* were substantially related to the flow of acetyl-CoA to the TCA cycle and a conversion between 2-oxoglutarate and glutamate. Our results have not provided the TCA cycle’s metabolites; further identification of pertinent metabolites or the application of integrative bioinformatic approaches might increase our knowledge of the actual mechanism. For PHB synthesis, it is important to note that all *pha* genes in the PHB synthetic pathway were increased in their transcript amounts under the BG_11_-N-P + A condition, in particular the *phaC* and *phaE* genes, in comparison to the normal BG_11_ condition (Fig. 7B, C). Nonetheless, our findings demonstrate a strong correlation between the increased amounts of *phaA* and *phaB* transcripts and the improved synthesis of PHB in the modified strains (OXP, Δ*adc1*c, and OXP/Δ*adc1*) (Fig. 8). This was in line with a previous study in *Synechocystis* sp. PCC6803, where increased PHB synthesis was associated with overexpression of the *phaAB* gene rather than *phaEC* overexpression during nitrogen deprivation [40]. On the other hand, the acetyl-CoA direction to fatty acid synthesis was also induced by the BG_11_-N-P + A condition due to the high upregulation of the *accA* transcript level and a slight induction of the *plsX* transcript (Fig. 7B, C). This could imply that acetate addition contributes to lipid production in cyanobacteria [40, 41]. According to Ref. [42], cyanobacterial cells grown in high C/low N conditions functioned by preventing the inhibitory interaction of PII protein with ACCase, while cells grown in high N/low C conditions could enhance the PII-ACCase interaction, leading to an inhibition of the ACCase enzyme.

## Conclusions

The nitrogen and phosphorus-deprived condition efficiently induced the accumulation of glycogen and PHB in *Synechocystis* sp. PCC 6803. In this study, higher PHB production was attained in three modified *Synechocystis* sp. PCC6803 strains, including Δ*adc1*c, OXP, and OXP/Δ*adc1*, under the nutrient-deprived treatments, in particular nitrogen and phosphorus-deprived BG_11_ medium with acetate addition (BG_11_-N-P + A). The *proC* overexpression and *adc1* knockout in *Synechocystis* apparently induced the changes in proline and glutamate contents inside the cells, which partially alleviated cells under nitrogen and phosphorus deprivation. However, the acetate addition, enhancing acetyl-CoA metabolite, significantly boosted the PHB and glycogen storage. These genetically modified strains of *Synechocystis* (Δ*adc1*c, OXP, and OXP/Δ*adc1*) might serve as practicable cell factories for biotechnological applications including biomaterials and biofuels.

## Materials and Methods

### Construction of *proC-*overexpressing *Synechocystis* sp. PCC 6803

First, the recombinant plasmid pEERM_*ProC* was constructed (Table 1), which was naturally transformed into *Synechocystis* sp. PCC 6803 wild type (WT) and Δ*adc1* mutant (obtained from [5]), thereby generating a *proC-*overexpressing *Synechocystis* sp. PCC 6803 (OXP), and an OXP lacking *adc1* gene (OXP/Δ*adc1*), respectively. The pEERM_*proC* plasmid was constructed by ligating the *proC* gene fragment amplified by PCR using a pair of ProC-F and ProC-R primers, as shown in Additional file 1: Table S1, in between the *Spe*I and *Pst*I restriction sites in the pEERM vector [26]. The correct recombinant plasmid pEERM_*proC* was transformed into WT and Δ*adc1* mutant cells by natural transformation to create OXP and OXP/Δ*adc1* strains, respectively. In addition, we also constructed the *Synechocystis* sp. PCC 6803 wild-type control (WTc) and the Δ*adc1* mutant control (Δ*adc1*c) by transforming the empty pEERM vector into WT and Δ*adc1* cells, represented as *Synechocystis* WT or Δ*adc1* mutant containing the *Cm*^*R*^ cassette gene (Fig. 2A). For host cell suspension preparation, the host cells (WT or Δ*adc1*) were cultures in BG_11_ medium until OD_730_ reaching about 0.3–0.5. Then, 10 mL of cell culture was harvested by centrifugation at 5500 rpm (3505 × *g*), 25 °C for 10 min, and cell pellets were resuspended in 500 μL of new BG_11_ medium. Next step, the host cell suspension was mixed with 10 μL of recombinant plasmid solution, and incubated that mixture overnight in the culture room with continuous light illumination at 40–50 μE/m^2^/s, at 28–30 °C. Then, the sample mixture was spread on a BG_11_ agar plate containing 10 μg/mL of chloramphenicol, and incubated in the culture room for 2–3 weeks until survived colonies occurred on plate. Each single colony was picked and streaked on a new BG_11_ agar plate containing higher concentrations of chloramphenicol (20 and 30 μg/mL), and incubated under same condition until transformant colonies appeared. The obtained transformants were confirmed for gene size, location, and segregation by PCR method using many specific pairs of primers (Additional file 1: Table S1).

### Strains and culture conditions

*Synechocystis* sp. PCC 6803 wild type (WT), derived from the Berkeley strain 6803 from fresh water in California, USA [43], *Synechocystis* lacking *adc1* gene (Δ*adc1*), and all engineered strains (WTc, Δ*adc1*c, OXP, and OXP/Δ*adc1*) were grown in normal BG_11_ medium for 16 days. The culture room, set for normal growth condition, was performed at 28–30 °C, with a continuous white light illumination by 40–50 μE/m^2^/s intensity. The cell culture flasks with the initial cell density at 730 nm (OD_730_) of about 0.05 were placed on the rotary shaker at 160 rpm speed. Cell growth was measured at OD_730_ by spectrophotometer. For nutrient-deprived conditions, all *Synechocystis* strains were initially grown in normal BG_11_ medium until late-log phase of cell growth before treating them with nutrient-derived media under the same growth condition for 11 days. There were two modified media including a BG_11_ medium without nitrogen (N) and phosphorus (P) (or BG_11_-N-P), and a BG_11_-N-P medium with 0.4%(w/v) acetate (A) addition (or BG_11_-N-P + A). For BG_11_-N-P medium, it was a BG_11_ medium lacking NaNO_3_ with KCl added in place of KH_2_PO_4_, and FeSO_4_ added in place of ferric ammonium citrate in equimolar concentrations [5]. In addition, the initial OD_730_ of cell culture under nutrient-modified conditions was adjusted to about 0.2. Acetate concentration in medium was determined according to the method of Ref. [44].

### Determinations of intracellular pigments and oxygen evolution rate

Cell culture (1 mL) was harvested by centrifugation at 12,000 rpm (14,383 × *g*) for 10 min. The intracellular pigments including chlorophyll *a* and carotenoids in cell pellets were extracted by N,N-dimethylformamide (DMF) (1 mL), vortexed and incubated under darkness for 10 min. After centrifugation at the same speed, the absorbance of the yellowish supernatant was spectrophotometrically measured at 461, 625, and 664 nm. The contents of chlorophyll *a* and carotenoids were calculated according to Refs. [45, 46]. For oxygen evolution rate, cell culture (10 mL) was harvested by centrifugation at 5500 rpm (3505 × *g*), 25 °C for 10 min. Cell pellets were resuspended in new BG_11_ medium (1 mL) and incubated under darkness for 30 min before measuring the oxygen evolution. Saturated light source was employed at 25 °C using Clark-type oxygen electrode (Hansatech instruments Ltd., King’s Lynn, UK). The unit of oxygen evolution rate was addressed as μmol O_2_/mg chlorophyll a/h [5].

### Total RNAs extraction and reverse transcription-polymerase reaction (RT-PCR)

Total RNAs were extracted from *Synechocystis* cells using the TRIzol® Reagent (Invitrogen, Life Technologies Corporation, Carlsbad, CA, USA). The purified RNAs (1 μg) were converted to cDNA by reverse transcription using ReverTra ACE® qPCR RT Master Mix Kit (TOYOBO Co., Ltd., Osaka, Japan). This obtained cDNA was subsequently used as a template for PCR with different pairs of primers (Additional file 1: Table S1). The PCR conditions were performed by initial denaturing at 95 °C for 5 min, followed by 30 cycles of 95 °C for 30 s, annealing temperature of each gene (Additional file 1: Table S1) for 30 s, and 72 °C for 35 s, followed by a final extension at 72 °C for 5 min. For *16s* rRNA reference, the PCR condition was the same, but there was 19 cycles instead. Prior to initiating the experiment, the optimum cycle for all genes was determined. Those bands were not saturated; instead, they were in the appropriate cycle. The PCR products were checked by 1.5% (w/v) agarose gel electrophoresis. Quantification of band intensity was detected by Syngene® Gel Documentation (Syngene, Frederick, MD, USA).

### HPLC analysis of PHB contents and Nile red staining

Cell cultures (50 mL) were harvested by centrifugation at 5500 rpm (3505 × *g*) for 10 min. To prepare sample for HPLC detection, cell pellets were hydrolyzed by boiling for 60 min with 98%(v/v) sulfuric acid (800 μL) and 20 mg/mL adipic acid (100 μL), an internal standard [5]. After that, the hydrolyzed sample was filtered using a 0.45 μm polypropylene membrane filter which was further detected by HPLC instrument (Shimadzu HPLC LGE System, Kyoto, Japan) using a carbon-18 column, Inert sustain 3-μm (GL Science, Tokyo, Japan), with a UV detector at 210 nm. The running buffer consisted of 30% (v/v) acetonitrile dissolved in 10 mM KH_2_PO_4_ (pH 7.4), with a flow rate of 1.0 mL/min. Authentic commercial PHB was used as standard, which was prepared as same as the samples. For dry cell weight (DCW), it was determined by incubating cell pellets in an oven at 80 °C for 16–18 h, until obtaining the constant weight.

For Nile red staining, cell culture (1 mL) was harvested by centrifugation at 5500 rpm (3505 × *g*) for 10 min. Cell pellets were resuspended in Nile red staining solution (3 μL). Then, the addition of normal saline (0.9%, w/v, 100 μL) was conducted and incubated overnight under darkness [5, 40]. To visualize the stained cells, fluorescent microscope (Carl Zeiss, Oberkochen, Jena, Germany) was applied using a filter cup with 535 excitation wavelength, at a magnification of 100x.

### Extraction and determination of glycogen content

Harvested cell pellets from liquid culture (15–30 mL) were extracted by alkaline hydrolysis [47, 48]. The 30% potassium hydroxide (KOH) solution (400 μL) was mixed with cell pellets, and boiled for 1 h. After centrifugation at 12,000 rpm (14,383 $\times$ g), 4 °C for 10 min, the supernatant was transferred to a new tube and mixed with 900 μL of cold absolute ethanol before incubating at − 20 °C overnight to precipitate glycogen. Next step, the sample mixture was centrifuged at 12,000 rpm (14,383 $\times$*g*), 4 °C for 30 min to obtain glycogen pellets which were subsequently dried at 60 °C overnight. Glycogen pellets were dissolved in 1 mL of 10% H_2_SO_4_. Then, dissolved sample (0.2 mL) was mixed with 10% H_2_SO_4_ (0.2 mL) and anthrone reagent (0.8 mL) before boiling for 10 min. After cooling down the samples to room temperature, the absorbance of samples was measured at 625 nm by spectrophotometer (modified from [4, 7]). A commercial oyster glycogen was used as the standard which was prepared as similar as the sample. The unit of glycogen content was %w/DCW.

### Extraction and determination of polyamine content

Total polyamines were extracted from *Synechocystis* cells with 5% cold HClO_4_ (modified from [49, 50]. After extraction by 5% cold HClO_4_ for 1 h on ice, the extracted samples were centrifuged at 12,000 rpm (14,838 $\times$ *g*) for 10 min. The supernatant and pellet fractions were represented as the fraction containing free and bound forms of polyamines, respectively. Both fractions were used to derivatize and quantify the total polyamines. For the derivatization, it was performed with benzoyl chloride using 1,6-hexanediamine as an internal standard. 1 mL of 2 M NaOH was mixed with 500 µL of HClO_4_ extract and 10 µL of benzoyl chloride. After vigorously mixing, the mixture was incubated for 20 min at room temperature. To terminate the reaction, saturated NaCl solution (2 mL) was added. The benzoyl polyamines were subsequently extracted by cold diethyl ether (2 mL). In addition, the ether phase (1 mL) was evaporated to dryness, and redissolved in methanol (1 mL). Authentic polyamine standards were prepared as similar as the samples. The polyamine content was detected by high-performance liquid chromatography (HPLC; Shimadzu HPLC LGE System, Kyoto, Japan) with inertsil®ODS-3 C-18 reverse phase column (5 μm; 4.6 × 150 mm) with UV–Vis detector at 254 nm. The mobile phase was a gradient of 60–100% methanol with a flow rate of 0.5 mL/min.

### Quantification of proline, glutamate, and GABA contents

HPLC detection of amino acids, including proline, glutamate, and GABA, was performed using *o*-phthalaldehyde (OPA) and 9-Fluorenylmethyl chloroformate (FMOC) derivatives (modified from [51, 52]). Cell pellets obtained from cell culture (50 mL) were washed and resuspended in 10 mM potassium phosphate citrate buffer (pH 7.6). Cell suspensions were homogenized using SONOPLUS Ultrasonic homogenizer (BANDELIN electronic GmbH & Co., Berlin, Germany). The supernatant was collected after centrifugation at 12,000 rpm (14,838 $\times$ g) for 10 min, and concentrated by a Centrivap concentrator (Labconco Corporation, MO, USA). The concentrated sample was further extracted with 600 μL of a mixture of water:chloroform:methanol (3:5:12,v/v/v), followed by 300 μL of chloroform and 450 μL of distilled water before centrifugation again at 5500 rpm (3505 × g), 4 ºC, for 10 min. The upper fraction of water–methanol phase was collected, and evaporated before redissolving in 200 μL of 0.1 N HCl. The sample solution was filtered through a 0.45 μm membrane filter, and then diluted (1:4,v/v) with internal standard solution of norvaline and sarcosine (62.5 mM in 0.1 M HCl). Then, this mixture was again filtered through a 0.45 μm membrane filter before detecting by HPLC with UV–VIS detector (Shimadzu HPLC LGE System, Kyoto, Japan) using 4.6 × 150 mm, 3.5 μm Agilent Zorbax Eclipse AAA analytical column and 4.6 × 12.5 mm, 5.0 μm guard column (Agilent Technologies, CA, USA). For the mobile phase, eluent A was 40 mM Na_2_HPO_4_, pH 7.8, and eluent B was acetonitrile:methanol:water (45:45:10, v/v/v), with a flow rate of 2 mL/min. The OPA- and FMOC-derivatized amino acids were monitored at 338 and 262 nm, respectively. The unit of amino acid content was nmol/mg protein.

### Supplementary Information

**Additional file 1: Table S1.** Primers used in this study. **Figure S1.** Agarose gel electrophoresis of RT-PCR products of proC transcript in Synechocystis sp. PCC 6803 strains grown under normal BG11 condition for 6 days, shown in Figure 2C. The 16s rRNA transcript was used as the reference (a size of 521 bp). ProC transcript size of 315 bp. **Figure S2.** Acetate concentration in BG11-N-P+A medium during adaptation phase of all strains. Cells were treated in BG11-N-P+A medium for 11 days. Medium was sampled at days 0, 1, 3, 5, 7, 9, and 11 for determining acetate concentration (according to the method of Hutchens and Kass, 1949). The error bars represent standard deviations of means (mean ± S.D., n = 3).

## Data Availability

Data generated and analyzed during this study are included in the published article.

## Abbreviations

ADC: Arginine decarboxylase
Arg: Arginine
Car: Carotenoids
Chl *a*: Chlorophyll* a*
DCW: Dry cell weight
DMF: N,N-Dimethylformamide
FMOC: 9-Fluorenylmethyl chloroformate
GABA: Gamma-aminobutyric acid
h: Hour
μg: Microgram
mL: Milliliter
min: Minute
nm: Nanometer
OD: Optical density
OPA: O-Phthalaldehyde
PCR: Polymerase chain reaction
PHB: Polyhydroxybutyrate
rpm: Revolutions per minute
s: Seconds
WT: Wild type
