# Supplementary material for: The adc1 knockout with proC overexpression in Synechocystis sp. PCC 6803 induces a diversion of acetyl-CoA to produce more polyhydroxybutyrate
Source: Biotechnol Biofuels Bioprod. 2024 Jan 13;17:6. doi: 10.1186/s13068-024-02458-9 (PMC10788017; doi:10.1186/s13068-024-02458-9)

Supplementary information

Table S1 Primers used in this study

Target gene	Name	Oligo sequences	Amplified fragment length (bp)	Annealing temperature
proC	ProC-F	5’-ggACTAGTTATCTCCCCGACTTAGCC-3’	1051	For PCR
	ProC-R	5’-aaCTGCAGAATCCCGTTCCTACCAAACC-3’		
Cm[R]	Cm[R]-F	5’-CTCGAGGCTTGGATTCTCTCAC-3’	900	For PCR
	Cm[R]-R	5’-CTCGAGGCTTGGATTCTCTCAC-3’		
psbA2	Up_psbA2-F	5’-CTTTAGCGTTCCAGTGGATATTTGC-3’	2360	For PCR
	Dw_psbA2-R	5’-TTGTAACGGGCGATCGCCTTGGCAA-3’		
proC	RT-ProC-F	5’- GGCTAAGGCTAAGGCCATCT-3’	315	50 °C
	RT-ProC-R	5’- CGAAAACCCATCTTCTCCAA-3’		
phaA	phaA-F	5’- CATGATGGTTTGACGGACAG-3’	310	50 °C
	phaA-R	5’- GACTACAGTTGCCCGCTGTT-3’		
phaB	RT-phaB-F	5’- ATGCCGGTATCACCAAAGA-3’	390	50 °C
	RT-phaB-R	5’- CAATTTCCTCCGGTTTACCA-3’		
phaC	RT-phaC-F	5’- GGGCACATTTAGCCTGTGTT-3’	346	50 °C
	RT-phaC-R	5’- GTAAGTTTCCCCCGCTTGAT-3’		
phaE	RT-phaE-F	5’- GAGCAATATACCGCCACCAC-3’	371	50 °C
	RT-phaE-R	5’-TCTTCCATCAAAGCAGCAAA -3’		
accA	RT-acc-F	5’- CGGGAAATGTTTCGGTTAGA -3’	415	55 °C
	RT-acc-R	5’- CCGCCGTTTCTAAAAATTGA-3’		
ach	RT-ach-F	5’- CTGGCGATCATCAAACAGCA -3’	281	60 °C
	RT-ach-F	5’-TCGTAAACCCCTTCGCTCAT -3’		
ack	RT-ack-F	5’- AGGCCAAAGCAGAGGGTAAT -3’	283	59 °C
	RT-ack-R	5’- AACACCGTCACCAAGGAGTC -3’		
acs	RT-acs-F	5’-CGGTTATTTTTGGGTCATGG -3’	339	62 °C
	RT-acs-R	5’-GAGGCTAAACTCCGCAACAG -3’		
gad	RT-gad-F	5’-CAGTGAAGCGGAAAGCCTAC-3’	352	60 °C
	RT-gad-R	5’- AGAACCAATGGTGGAACAGC -3’		
gdhA	RT- gdhA -F	5’- GCGTTTAAGTCGGGGTTACA -3’	482	61 °C
	RT- gdhA -R	5’-TTTCCCCTAAATCGCAGATG -3’		
gltA	RT- gltA -F	5’- ATGAATGCGTCCACCTTTTC -3’	381	52 °C
	RT- gltA -R	5’- GTCCACATTGGGGTAAATGC-3’		
glgX	RT- glgX -F	5’- GAGCTTCATCGAGGACGGAA- 3’	360	64 °C
	RT- glgX -R	5’- GCCCGAATTGGGGTTGCGGG - 3’		
plsX	RT- plsX -F	5’- AAGGGGTGGTGGAAATGGAA - 3’	467	58 °C
	RT- plsX -R	5’- AAGTACGTCCCTTCCTTCGG - 3’		
putA	RT-putA -F	5’- GTGATTTCCCCCTGGAATTT - 3’	414	56 °C
	RT-putA -R	5’- ACCAAAGGCGGAATACACTG - 3’		
16s rRNA	RT-16 -F	5’- AGTTCTGACGGTACCTGATGA - 3’	521	55°C
	RT-16s-R	5’- GTCAAGCCTTGGTAAGGTTCT - 3’		

Figure S1

Agarose gel electrophoresis of RT-PCR products of *proC* transcript in *Synechocystis sp.* PCC 6803 strains grown under normal BG_{11} condition for 6 days, shown in **Figure 2C**. The *16s* rRNA transcript was used as the reference (a size of 521 bp). *ProC* transcript size of 315 bp.

***ProC* gene : OXP strain**

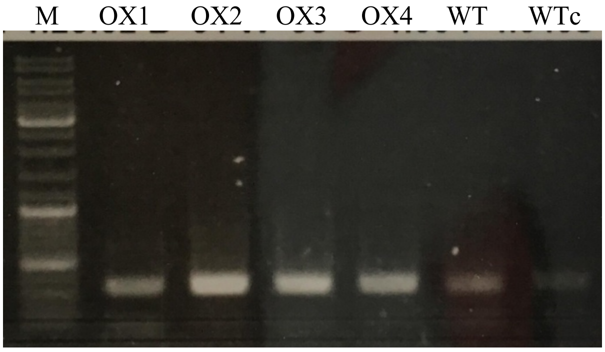

***16s* rRNA gene : OXP strain**

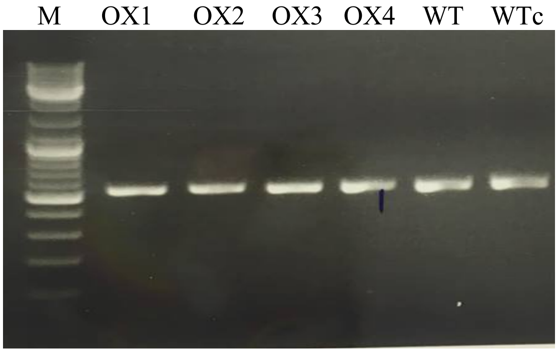

***ProC* gene : OXP/Δ*adc1* strain**

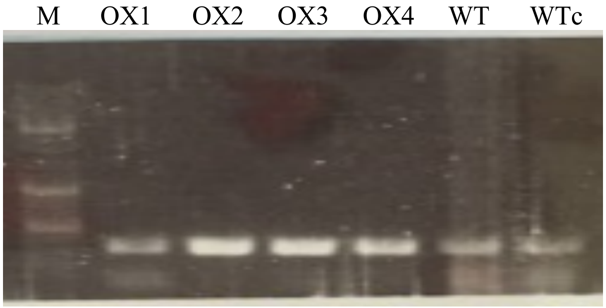

***16s* rRNA gene : OXP/Δ*adc1* strain**

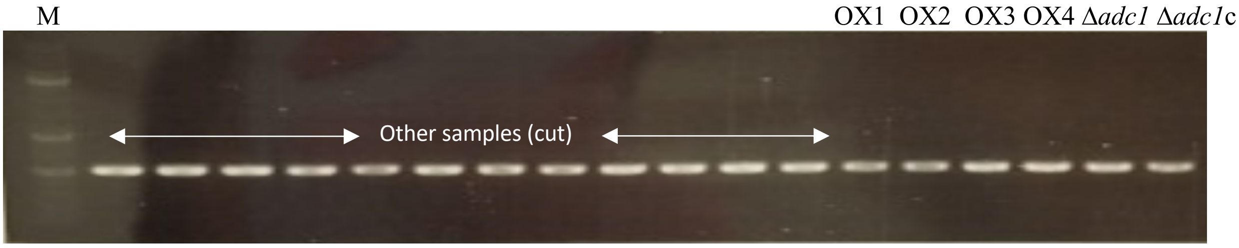

Figure S2 Acetate concentration in BG_{11}-N-P+A medium during adaptation phase of all strains. Cells were treated in BG_{11}-N-P+A medium for 11 days. Medium was sampled at days 0, 1, 3, 5, 7, 9, and 11 for determining acetate concentration (according to the method of Hutchens and Kass, 1949). The error bars represent standard deviations of means (mean ± S.D., n = 3).

A) The reaction mixture : BG_{11}-N-P and BG_{11}-N-P+A media

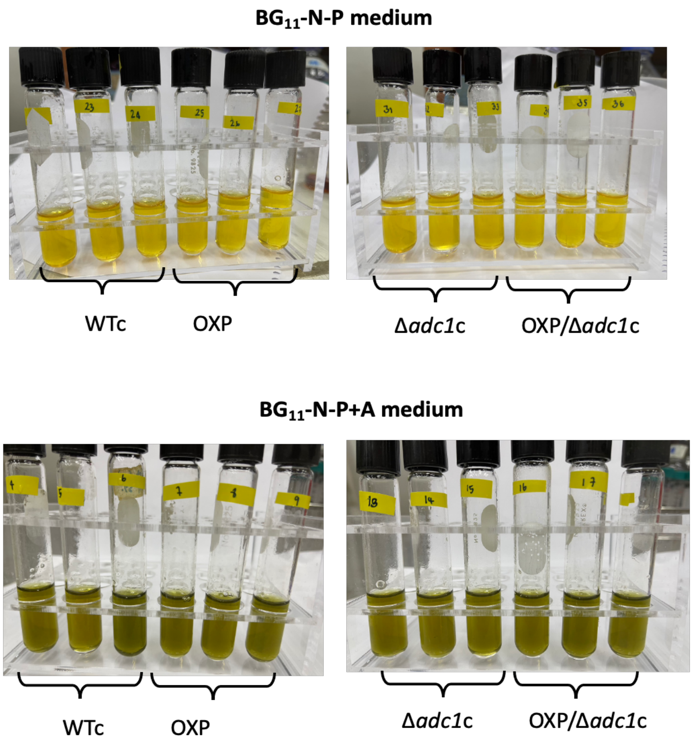

B) Acetate concentration in medium during adaptation phase (0.4% acetate = 48 mM)

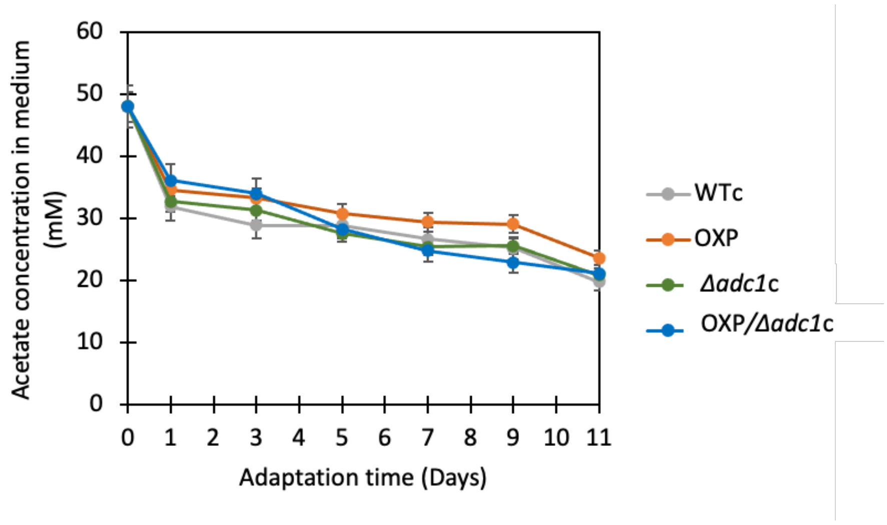

Supplement: Supplementary file 1 — Additional file 1: Table S1. Primers used in this study. Figure S1. Agarose gel electrophoresis of RT-PCR products of proC transcript in Synechocystis sp. PCC 6803 strains grown under normal BG11 condition for 6 days, shown in Figure 2C. The 16s rRNA transcript was used as the reference (a size of 521 bp). ProC transcript size of 315 bp. Figure S2. Acetate concentration in BG11-N-P+A medium during adaptation phase of all strains. Cells were treated in BG11-N-P+A medium for 11 days. Medium was sampled at days 0, 1, 3, 5, 7, 9, and 11 for determining acetate concentration (according to the method of Hutchens and Kass, 1949). The error bars represent standard deviations of means (mean ± S.D., n = 3). [file 13068_2024_2458_MOESM1_ESM.pdf]
